# Emerging role of the host microbiome in neuropsychiatric disorders: overview and future directions

**DOI:** 10.1038/s41380-023-02287-6

**Published:** 2023-10-16

**Authors:** Kenji Hashimoto

**Affiliations:** Division of Clinical Neuroscience, Chiba University Center for Forensic Mental Health, Chiba, 260-8670 Japan

**Keywords:** Depression, Neuroscience

## Abstract

The human body harbors a diverse ecosystem of microorganisms, including bacteria, viruses, and fungi, collectively known as the microbiota. Current research is increasingly focusing on the potential association between the microbiota and various neuropsychiatric disorders. The microbiota resides in various parts of the body, such as the oral cavity, nasal passages, lungs, gut, skin, bladder, and vagina. The gut microbiota in the gastrointestinal tract has received particular attention due to its high abundance and its potential role in psychiatric and neurodegenerative disorders. However, the microbiota presents in other body tissues, though less abundant, also plays crucial role in immune system and human homeostasis, thus influencing the development and progression of neuropsychiatric disorders. For example, oral microbiota imbalance and associated periodontitis might increase the risk for neuropsychiatric disorders. Additionally, studies using the postmortem brain samples have detected the widespread presence of oral bacteria in the brains of patients with Alzheimer’s disease. This article provides an overview of the emerging role of the host microbiota in neuropsychiatric disorders and discusses future directions, such as underlying biological mechanisms, reliable biomarkers associated with the host microbiota, and microbiota-targeted interventions, for research in this field.

## Introduction

According to the Global Burden of Diseases, Injuries, and Risk Factors Study (GBD) 2019, neuropsychiatric disorders such as schizophrenia, autism spectrum disorder (ASD), major depressive disorder (MDD), bipolar disorder (BD), anxiety disorders, and substance use disorder, continue to rank among the top ten leading causes of global burden, with no evidence of a reduction since 1990 [1]. These disorders have profound impacts on individuals, their families, and communities, posing a significant public health concern worldwide. Additionally, the prevalence of MDD, anxiety disorders, and post-COVID-19 condition has increased during and following the COVID-19 pandemic [2–9]. Accumulating evidence indicates that both genetic and environmental factors contribute significantly to the development and manifestation of these neuropsychiatric disorders [10–18]. Environmental factors encompass early life experiences, social and cultural factors, traumatic events, chronic stress, substance abuse and addiction, as well as limited access to mental healthcare services. However, the precise biological mechanisms underlying the development and progression of neuropsychiatric disorders remain elusive.

Brain–body crosstalk constitutes a bidirectional network that facilitates the regulation and maintenance of overall homeostasis in the body by enabling communication between the brain and peripheral organs [19–30]. This crosstalk involves various components, including the central nervous system (CNS), peripheral nervous system, neurotransmitters, chemical signaling, hormones, feedback loops and homeostasis, and mind–body connection associated with emotions. A comprehensive understanding of brain–body crosstalk is crucial for scientists to unravel the underlying mechanisms contributing to the pathogenesis of neuropsychiatric disorders.

**Fig. 1:**
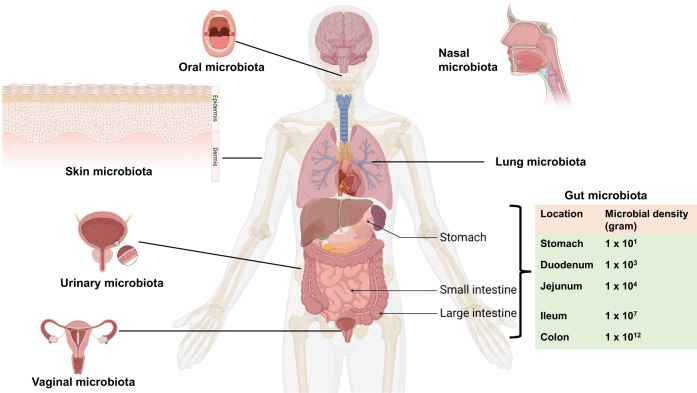
Host microbiota in human body. The human microbiota resides in the various tissues of the body, including the mouth, nose, gastrointestinal (GI) tract, lung, skin, bladder, and vagina. In the GI trats, the density of microbes in different locations, such as the stomach, duodenum, jejunum, ileum, and colon, has been shown [43]. Part of the figure was designed using resources from Biorender.com.

The human body is known to harbor a diverse and abundant community of microorganisms called the microbiota. Host microbiota can be categorized into various types, including oral, nasal, lung, gut, skin, bladder, and vagina microbiota (Fig. 1) [31–35]. The gut microbiota, which resides in the gastrointestinal (GI) tract, has garnered increasing attention due to its role in brain–body crosstalk, known as the gut–brain axis [20, 28, 29, 36–40]. However, limited research has been conducted on the role of other host microbiota in neuropsychiatric disorders due to their lower abundance compared to the gut microbiota. It is essential to comprehensively understand the role of the predominant gut microbiota as well as other microbiota in neuropsychiatric disorders.

In this article, the author provides an overview of the host microbiota in humans and explores the emerging role of the host microbiota in neuropsychiatric disorders. Furthermore, the author proposes future research directions for investigating the role of the host microbiota in neuropsychiatric disorders.

## Host microbiome in human

**Fig. 2:**
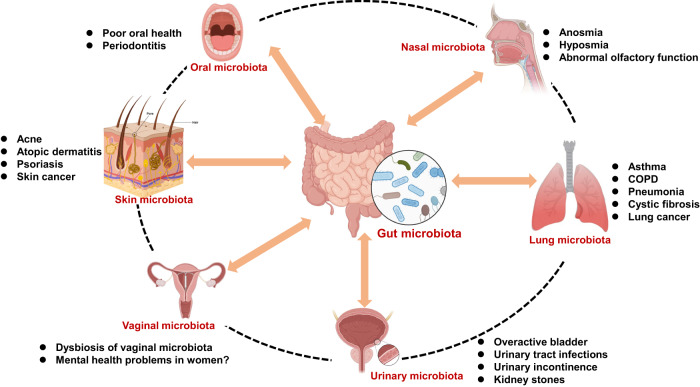
Potential interactions among the microbiota in the different organs. The microbiota in one organ may potentially influence that in another organ. The gut microbiota, which constitutes the majority of the host’s microbiome, plays a central role in affecting health and disease throughout the body. Although there are proposed interactions between the microbiota of different organs, current evidence supporting these interactions remains limited. Dysbiosis in the microbiota across various different tissues may contribute to the incidence of organ-specific diseases. COPD: chronic obstructive pulmonary disease. Part of the figure was designed using resources from Biorender.com.

The role of the host microbiota in maintaining health and contributing to various diseases has gained considerable attention. The host-microbiota encompasses a range of microbial communications, including the oral, nasal, lung, gut, skin, bladder, and vaginal microbiota (Fig. 1) [31, 34, 35]. Notably, the oral and nasal microbiota serve as crucial entry points for potential pathogens that could spread to the CNS. Human microbial communities are complex and interconnected (Fig. 2). Microbiota in one organ have the potential to influence those in another. The gut, which houses the majority of the host’s microbiome, plays a central role in affecting overall health and influencing various diseases throughout the body [31, 34, 41, 42]. Although potential interactions among microbiota in different organs have been suggested, empirical evidence to support these claims remains limited (Fig. 2). Current research is focused on elucidating these microbial interactions across various organs.

While numerous studies have focused on the gut microbiota in the GI tract, research on other microbiota has been limited due to their lower abundance compared to the gut microbiota [43]. In the next sections, the author provides a summary of the role of the host microbiota in the GI tract and the other different tissues.

## Gut microbiota

The density (gram) of microbiota in the GI tract varies across different regions: stomach (1 × 10^1^), duodenum (1 × 10^3^), jejunum (1 × 10^4^), ileum (1 × 10^7^), and colon (1 × 10^12^) (Fig. 1) [43]. The composition and diversity of gut microbiota in these different regions may differ across these regions; however, studying the gut microbiota from fecal samples, which are closely related to the colon, is more straightforward. The term “gut microbiota–brain axis” refers to the bidirectional communications between the gut microbiota in the GI tract and the brain. Accumulating evidence strongly suggests that the gut microbiota plays a crucial role in regulating brain function and behavior through various mechanisms. These mechanisms include the production of various neurotransmitters, including serotonin, dopamine, γ-aminobutyric acid (GABA), kynurenic acid, as well as microbe-derived metabolites (e.g., short-chain fatty acids [SCFAs], bile acids, D-amino acids). With regard to D-amino acids, some studies have demonstrated reduced blood levels of D-glutamate in patients with Alzheimer’s disease (AD) compared to healthy controls [44, 45]. Moreover, it has been observed that plasma levels of D-glutamate were correlated with cognitive functions [44, 45]. Given that D-glutamate is a component of the peptidoglycan cell wall in bacteria, it is plausible that the gut microbiota contributes to its production [46]. The immune system also plays a role in this axis [20, 28, 29, 47]. Preclinical findings have highlighted the significance of the vagus nerve in the gut microbiota–brain axis [48–57]. Understanding the gut microbiota–brain axis via the vagus nerve has opened up new possibilities for the development of novel treatments (e.g., dietary interventions, prebiotics, probiotics, symbiotics [synergistic combination of prebiotics and probiotics], fecal microbiota transplantation, and vagus nerve stimulation) for neuropsychiatric disorders [9, 39, 40, 58].

Research on the gut microbiota–brain axis in neuropsychiatric disorders is a rapidly evolving field. Alterations in the composition and diversity of the gut microbiota have been linked to various psychiatric disorders, including schizophrenia, MDD, BD, ASD, anxiety, and even neurodegenerative disorders such as AD and Parkinson’s disease (PD) [59–71]. A meta-analysis identified a transdiagnostic pattern associating gut microbiota imbalances with schizophrenia, MDD, BD, and anxiety [72]. These imbalances were characterized by a reduction of certain anti-inflammatory butyrate-producing bacteria and an increase in pro-inflammatory bacteria [72]. Specifically, consistent reductions in the levels of *Faecalibacterium* and *Coprococcus*, along with elevated levels of *Eggerthella*, were observed across psychiatric disorders such as schizophrenia, MDD, BD, and anxiety [72]. A separate systematic review highlighted bacterial taxa frequently linked to psychiatric disorders (e.g., schizophrenia, MDD, BD) [64]. These findings include reduced levels of bacterial genera that produce SCFAs (e.g., butyrate), increased levels of lactic acid-producing bacteria, and a heightened presence of bacteria involved in the metabolism of neurotransmitters such as glutamate and GABA [64].

A meta-analysis, which included discovery and replication samples, confirmed that ten bacterial genera were significantly correlated with AD [73]. Among these, four genera were significantly associated with the APOE rs429358 risk allele, either as protective or risk factors for AD. Importantly, the pro-inflammatory genus *Collinsella*, identified as a risk factor for AD, exhibited a positive correlation with the APOE rs429358 risk allele across both sample sets. These findings indicate that the influence of host genetic factors on the abundance of these ten genera is significantly correlated with AD, suggesting that these genera could serve as potential biomarkers and therapeutic targets for the disease [73]. Another recent meta-analysis showed that, at the phylum level, the relative abundance of *Firmicutes* was significantly lower in AD patients compared to healthy controls [74]. Conversely, the relative abundance of *Bacteroidetes* was significantly higher in patients with MCI than in healthy controls [74]. Collectively, these findings highlight gut microbiota abnormalities associated with AD.

PD is a neurodegenerative disorder primarily characterized by motor symptoms, including tremors, rigidity, bradykinesia, and postural instability. Notably, a significant number of PD patients experience GI symptoms, such as constipation, well before the onset of motor symptoms. This suggests that alterations in gut motility could be linked to gut microbiota dysbiosis. A protein named α-synuclein accumulates in the brains of PD patients, forming aggregates termed Lewy bodies. Interestingly, these protein aggregates are also found in the enteric nervous system (the nerve of the gut). Current hypotheses suggest that pathologic α-synuclein may originate in the gut and subsequently migrate to the brain via the vagus nerve, thereby contributing to the pathology of PD [60, 71, 75–78].

However, it is important to note that the gut microbiota–brain axis is a complex and evolving field of research. Further studies are needed to elucidate the mechanisms and develop new effective therapies. Nonetheless, the emerging evidence underscores the significant role of the gut microbiota in psychiatric and neurodegenerative disorders, offering a new perspective on the treatment and management of these disorders. The author has not extensively explored the role of gut microbiota on neuropsychiatric disorders in this section, as numerous comprehensive review articles have already been published [64–71].

## Oral microbiota

**Fig. 3:**
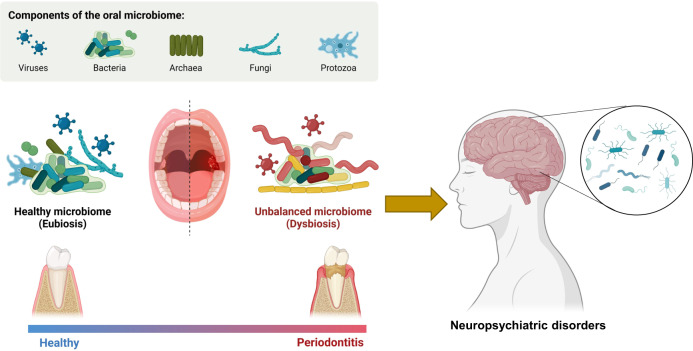
Role of oral microbiota in neuropsychiatric disorders. The oral microbiota consists viruses, bacteria, archaea, fungi, and protozoa. In comparison to a healthy microbiome (eubiosis), patients with periodontitis exhibit an imbalanced microbiome (dysbiosis), which can contribute to the development of neuropsychiatric disorders. Studies utilizing postmortem brain samples have demonstrated the presence of the oral microbiota in the brains from patients with Alzheimer’s disease (AD) or Parkinson’s disease [105]. Part of the figure was designed using resources from Biorender.com.

The oral microbiota refers to the collective microbial community inhabiting the human oral cavity. It represents the second-largest microbial community in the human body. Components of the oral microbiome are viruses, bacteria, archaea, fungi, and protozoa (Fig. 3) [34, 35, 79, 80]. The oral microbiota colonizes two distinct regions: the hard surfaces of the teeth, including dentures, and the soft tissues of the oral mucosa. Major phyla of the oral microbiota include *Actionobacteria, Bacteroidetes*, *Firmicutes*, *Fusobacteria*, and *Proteobacteria* (Table 1) [34, 35]. Considering that the oral cavity serves as the primary entry point to the human body, disruptions in the oral microbiota may potentially contribute to the development and progression of psychiatric and neurodegenerative disorders, as well as autoimmune diseases (Fig. 3) [34, 35, 81–83]. In addition of the gut microbiota, research efforts have expanded to investigate the role of the oral microbiota in neuropsychiatric disorders [84–89].

**Table 1:** Predominant microbiota in the different sites of human body.

Tissue	Predominant bacterial phyla
Mouth	*Actinobacteria, Bacteroidetes, Firmicutes, Fusobacteria, Proteobacteria*
Nose	*Actinobacteria, Firmicutes, Proteobacteria*
Stomach	*Actinobacteria, Bacteroidetes, Firmicutes, Fusobacteria, Proteobacteria*
Intestine	*Bacteroidetes, Firmicutes*
Lung	*Actinobacteria, Bacteroidetes, Firmicutes, Proteobacteria*
Skin	*Actinobacteria, Bacteroidetes, Firmicutes, Proteobacteria*
Bladder	*Firmicutes*
Vagina	*Firmicutes (the species Lactobacillus)*

A slight modification from the references [34, 35].

### Periodontal diseases in patients with psychiatric disorders

Patients with psychiatric disorders often exhibit poor oral hygiene and a compromised periodontal status [84]. A meta-analysis has indicated that all psychiatric disorders are associated with an increased risk of dental decay, as reflected by higher decayed, missing, and filled teeth scores, as well as greater tooth loss [90]. Dry mouth, a common side effect of medications, is prevalent among many individuals with neuropsychiatric disorders and serves as a significant risk factor for oral health issue [84]. Furthermore, the incidence of periodontitis in patients with psychiatric disorders is 1.45 times higher compared to those without psychiatric disorders [91]. Taken together, these findings suggest that periodontitis may partly contribute to the risk for developing neuropsychiatric disorders (Figs. 2 and 3). However, further detailed studies with larger sample sizes are needed.

In a case-control study involving BD patients (*n* = 176) and controls (*n* = 176), the prevalence of periodontitis was higher among BD patients, and BD patients with periodontitis exhibited elevated levels of *Aggregatibacter actinomycetemcomitans*, and *Porphyromonas gingivalis* compared to controls [92]. Notably, periodontitis showed a strong association with both the total bacterial load and the depressive phase of BD [92]. These findings suggest that increased levels of these oral microbiota may contribute to periodontitis, potentially leading to depressive symptoms in BD patients. Additionally, the salivary microbiome in patients (*n* = 85) with drug-naïve first-episode schizophrenia was characterized by higher α-diversity (a measure of microbiome diversity) and lower β-diversity (a measure of the similarity or dissimilarity of microbial communities) heterogeneity than those of subjects (*n* = 43) with clinical high risk for psychosis and healthy controls (*n* = 80) [93]. Interestingly, hydrogen sulfide (H_2_S)-producing bacteria exhibited disease-stage-specific enrichment, and certain salivary microbiota exhibited disease-specific correlation patterns with symptom severity [93]. This supports previous findings highlighting the role of excess H_2_S in schizophrenia [94]. The same group reported changes in salivary metabolites in patients with drug-naïve first-episode schizophrenia compared to healthy controls, with these changes being closely associated with peripheral inflammatory markers and salivary microbiota [95]. These results suggest a connection between the disturbed oral microbiota, microbe-derived metabolites and the onset of schizophrenia, hinting at a possible role of the oral–brain connection in the initiation of this disease. However, further research involving larger sample sizes is necessary to confirm these findings.

A recent narrative review proposes a possible link between the oropharyngeal microbiome and schizophrenia, although additional research is needed to definitively establish this connection [96]. Another recent study showed that alcohol consumption influences the diurnal fluctuations in the oral microbiota of individuals with functional impairment due to alcohol dependence. This finding emphasizes the potential for interventions to mitigate the negative consequences of alcohol dependence [97].

Using a cohort of Israel veterans from the 1982 Lebanon war, Levert-Levitt et al. [98] found that the oral microbiota signature (specifically, reduced levels of bacteria *sp_HMT_914, 332* and *871*, as well as *Noxia*) correlated with the severity of post-traumatic stress disorder (PTSD). Conversely, the duration of education was associated with higher levels of *sp_HMP_871* and decreased levels of *Bacteroides* and *Firmicutes*. Notably, air pollution positively correlated with PTSD symptoms, psychopathological symptoms, and changes in oral microbiota composition [98]. These findings suggest potential non-intrusive treatments for PTSD related to the oral microbiota pathway.

### Neurodegenerative disorders and detection of bacteria in the brain

Mild cognitive impairment (MCI) represents an initial phase of memory decline or other cognitive functions, yet individuals with MCI retain the capability to conduct most daily activities. In subjects with MCI, higher levels of *Pasteurellacae* were observed compared to cognitively normal controls, whereas the abundance of *Lautropia mirabilis* was lower in individuals with MCI [99]. Furthermore, the abundance of *Pasteurellacae* was associated with inflammatory markers in the cerebrospinal fluid (CSF). These preliminary findings suggest that an altered composition of the oral microbiota may contribute to neuroinflammation, potentially leading to cognitive decline. However, further longitudinal studies involving elderly individuals are needed to better understand this relationship.

Both AD and PD are prominent neurodegenerative disorders, characterized by the accumulation of β-amyloid and α-synuclein in the brain, respectively. Patients with AD or PD often exhibit psychiatric symptoms, such as depression. A well-established association exists between poor oral health, specifically periodontitis, and an increased risk of developing AD or PD (Fig. 3) [100–102]. A cross-sectional study has revealed links between oral health-related stressors and neuropsychiatric symptoms in patients with AD [103]. Collectively, these findings highlight the importance of managing oral health in patients with neurodegenerative disorders.

Several microorganisms have been identified in the CSF and brains of individuals with AD or PD, suggesting a potential role in the progression of these diseases [104–106]. A recent study using the postmortem brain samples revealed the widespread presence of oral bacteria in regions associated with AD and PD pathology (Fig. 3). Interestingly, bacteria profiles in the brain were distinct from those in blood samples [107]. Additionally, a recent meta-analysis demonstrated a significant association between oral bacteria and AD, particularly when oral bacteria were detectable in the brain [108]. A recent study showed that the oral and gut microbiota of partners of AD patients resembled that of the AD patients themselves and differed from healthy controls [109], suggesting a potential transmission of microbiota. This observation could provide insight into why spouses of AD patients have an elevated risk of developing dementia [109]. Overall, it is plausible that periodontal microbiota could enter the brain, thereby contributing to the development of AD [110]. However, further cohort studies involving larger sample sizes are necessary to confirm the role of the oral microbiota in AD or PD. At present, it remains unknown whether oral bacteria are detected in the brains of patients with psychiatric disorders.

Increasing evidence highlights the role of oral microbiota in PD [111]. Notably, the oral microbiota in early-stage PD patients shows significant differences compared to healthy controls, with specific oral bacteria exhibiting associations with motor and non-motor functional measures [112]. Furthermore, significant disparities were observed in the composition of the oral and gut microbiome between PD patients and healthy controls [113]. Notably, the oral bacteria *Lactobacillus* demonstrated increased abundance in PD patients and was associated with opportunistic pathogens in the gut. Another study revealed significant differences in microbiota composition in the oral cavity and gut, but not the nasal cavity, between PD patients (*n* = 91) and healthy controls (*n* = 91) [114]. Interestingly, correlations between the genera in the oral cavity and the severity of depression and anxiety were observed in PD patients [114], suggesting a role of the oral microbiota in psychiatric symptoms of PD. These findings indicate a potential link between the oral and the gut microbiota in PD (Fig. 2), which could lead to functional changes within the microbiome of PD patients [113, 114]. Given the crucial role of oral microbiota in maintaining oral health, it is plausible that changes in the oral microbiota among the elderly population could contribute to the development and progression of various disorders such as AD, PD, and age-related systemic disorders [115, 116].

## Nasal microbiota

**Fig. 4:**
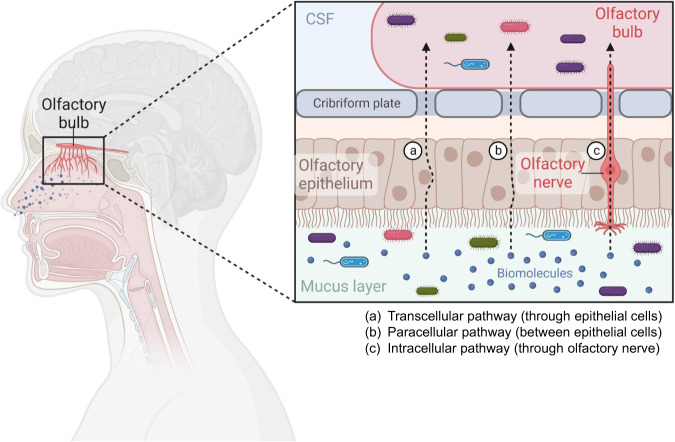
Nasal microbiota in the olfactory function. The nasal cavity can be divided into distinct regions, including the nasal vestibules, respiratory region, olfactory region, and nasopharyngeal region. The nasal microbiota colonizes these regions, and plays a crucial role in maintaining the health of the nasal mucosa and overall immune system function. There are three pathways from the mucus layer to the olfactory bulb: (a) the transcellular pathway, which involves passage through epithelial cells; (b) the paracellular pathway, which occurs between epithelial cells; and (c) the intracellular pathway, which occurs through the olfactory nerve. Considering the crucial role of the nasal microbiota within the nasal cavity, it is plausible that both the nasal microbiota and their metabolites may have a role in neuropsychiatric disorders. Part of the figure was designed using resources from Biorender.com.

The nasal cavity harbors a diverse community of microorganisms that contribute to the maintenance of the nasal mucosa health and overall immune system function [117–119]. Major phyla observed in the nasal microbiota include *Actionobacteria, Firmicutes*, and *Proteobacteria* (Table 1) [34, 35]. The composition and diversity of the nasal microbiota vary among individuals and are influenced by factors such as age, genetics, environmental exposures, and personal hygiene habits such as smoking [34, 117, 119]. Considering the role of the nasal microbiota in host immune responses, an imbalance in the nasal microbiota has been associated with various health conditions [34, 117, 119]. The role of the nasal microbiota in neuropsychiatric disorders is an emerging area of research that explores the potential link between the microbial communities in the nasal cavity and mental health conditions. Additionally, the nasal microbiota may influence the well-known gut microbiota–brain axis through various pathways, including direct contact with the olfactory system, immunes system modulation, and the production of neurotransmitters or metabolites capable of crossing the blood-brain barrier (Fig. 4). However, there are currently no reports available on the specific role of the nasal microbiota in patients with psychiatric disorders.

### Neurodegenerative disorders

Age significantly influences olfactory dysfunction, making it a potential early indicator of neurodegenerative disorders [119]. Anosmia (complete loss of olfactory function) and hyposmia (decreased olfactory function) are commonly observed in patients with neurodegenerative disorders. Although there is currently no direct evidence supporting the association between the inflammatory response of the nasal microbiota and neurodegenerative disorders, several reports explore the role of the nasal microbiota in AD and PD [119]. These studies suggest a potential link between microbial communities in the nasal cavity and the development or progression of these neurological disorders. Additionally, a potential association between PD and nasal microbiota has been proposed. Dysbiosis of the nasopharyngeal microbiota could trigger inflammatory responses to α-synuclein, contributing to the pathological changes seen in PD [119].

### Nasal microbiota in the olfactory function

Accumulating evidence suggests the involvement of olfactory dysfunction in neuropsychiatric disorders (Fig. 2). Specific alterations in various components of the sense of smell have been observed in patients with neuropsychiatric disorders such as schizophrenia [120–126]. However, there are currently no reports available on the relationship between the nasal microbiota and olfactory functions in patients with neuropsychiatric disorders. Notably, patients with substance use disorder often use inhalation of drugs of abuse. It is known that the respiratory system, including the nasal cavity and lungs, can be exposed to various drugs through inhalation, resulting in dysbiosis of nasal and lung microbiota. Given the crucial role of the nasal microbiota in olfactory functions, it is of great interest to investigate whether the nasal microbiota is altered in patients with neuropsychiatric disorders.

## Lung microbiota

The human respiratory tract was traditionally believed to be a sterile environment; however, emerging research using advanced techniques has revealed the presence of a diverse community of microorganisms known as the lung microbiota. Major phyla observed in the lung microbiota include *Bacteroidetes, Firmicutes*, and *Proteobacteria* (Table 1) [34, 35]. Various factors, including environmental exposers, host genetics, immune function, and lifestyle, influence the composition of the lung microbiota, highlighting its potential role in maintaining lung health and immune function [127–129]. Notably, alterations in the lung microbiota have been associated with respiratory diseases such as asthma, chronic obstructive pulmonary disease, pneumonia, cystic fibrosis, and lung cancer (Fig. 2) [127–131]. Interestingly, a gut–lung axis has been described, indicating crosstalk between the microbiomes of the gut and lungs (Fig. 2) [132, 133].

Currently, there is a limited body of research specifically focusing on the role of the lung microbiota in neuropsychiatric disorders. The lung–brain axis remains underexplored, although three potential mechanisms have been proposed [133]. First, microorganisms or their by-products might directly translocate across the capillary barrier into the bloodstream, eventually reaching the brain. Second, the lung microbiome could influence systemic humoral factors, given its role in local pulmonary immune homeostasis. Third, the lung microbiome may affect systemic cell-mediated immunity, which could subsequently impact brain function [133]. A recent prospective randomized study demonstrated that traumatic brain injury patients who developed ventilator-associated pneumonia during their ICU stay exhibited distinct structures of bronchoalveolar lavage microbiota both at admission and at seven days post-ICU admission [134]. This finding suggests the potential utility of lung microbiota management as a strategy for preventing infections in critically ill patients [134].

Additionally, a recent preclinical study demonstrated that antibiotic-induced disruption of the lung microbiome significantly increased the susceptibility of rats to developing autoimmune diseases in the CNS, suggesting the potential role of the lung microbiome on brain immune responses via the lung–brain axis [135]. Furthermore, antibiotic-induced microbiome depletion could reduce acute lung injury after lipopolysaccharide administration [136]. Another recent study demonstrated that small intestinal γδ T-cell migration into the lung and brain plays a role in stroke-associated pneumonia in mice [56]. Given the emerging recognition of the lung–brain axis [135, 137, 138], it becomes increasingly compelling to explore whether the lung microbiota plays a role in the development and progression of neuropsychiatric disorders.

## Skin microbiota

Human skin is home to millions of bacteria, fungi and viruses that compose the skin microbiota. As the largest organ of the human body, the skin microbiota plays essential roles in the protection against the invasion of pathogens (Fig. 1) [139, 140]. The composition of the skin microbiota varies across different body regions (e.g., glabella, sebaceous, antecubital fossa, volar forearm, toe web space) and among individuals, influenced by the factors such as age, genetics, hygiene practices, and environmental exposures [139–142]. Major phyla of the skin microbiota include *Actionobacteria, Bacteroidetes*, *Firmicutes*, and *Proteobacteria* (Table 1) [34, 35]. Given the important role of the skin microbiota in maintaining skin health and function, it is likely that the skin microbiota contributes to the regulation of the skin’s immune response, influencing inflammation and defense mechanisms. Disruptions in the skin microbiota can lead to various skin conditions and diseases. For example, an overgrowth of certain bacteria, such as *Staphylococcus aures*, has been associated with skin disorders such as acne, atopic dermatitis, and wound infections (Fig. 2) [139, 140].

The skin microbiota primarily influences the skin health; however, emerging research suggests a potential role of the skin microbiota in neuropsychiatric disorders due to its potential to influence the gut microbiota (Fig. 2) [34, 143, 144]. Therefore, it is possible that the skin microbiota may indirectly impact the gut–brain axis, leading to the development of neuropsychiatric disorders. Currently, there is limited research reporting alterations in the skin microbiota in patients with neuropsychiatric disorders. To the best of our knowledge, there is only one report that has reported alterations in the skin microbiota in patients with anorexia nervosa. Hermes et al. [145] identified significant correlations between Shannon diversity, the highly abundant skin antimicrobial peptide psoriasin, and bacterial load. Additionally, psoriasin was associated with *Abiotrophia*, an indicator for the healthy-weight control group. A significant correlation was observed between an individual’s body mass index and *Lactobacillus*, which serves as a microbial indicator of health. Further investigations examining the relationship between caloric and nutritional intake and the skin microbiota in patients with eating disorders are required to clarify the association between dietary factors and skin physiology [145]. In a recent study, Arikan et al. [146] demonstrated an association between axillary microbiota and cognitive impairment in PD patients (*n* = 103), suggesting a possible role of skin microbiota in cognitive impairments. Nonetheless, further studies with larger sample sizes are necessary to validate these findings.

Depression is a common psychiatric symptom in patients with skin diseases, such as psoriasis [147, 148]. Various studies have proposed the existence of a skin–microbiota–brain axis in the comorbidity of depression in patients with chronic wound [149–151]. Furthermore, alterations in both the skin and gut microbiota are believed to contribute to the pathogenesis of psoriasis through inflammatory and immune mechanisms [152]. A preclinical study using imiquimod-treated mice (a model of psoriasis) demonstrated correlations between the skin microbiota and the gut microbiota, suggesting bidirectional communication between the two [153]. Given the interaction between the skin microbiota and the gut microbiota in the immune system, it is intriguing to investigate whether skin microbiota is altered in patients of neuropsychiatric disorders.

## Bladder microbiota

Bladder (or urinary) microbiota has been identified in the human urinary tract. The abundance and diversity of the urinary microbiota are distinct from the microbiota of other body sites such as the gut or the skin. Major phylum of the bladder microbiota is *Firmicutes* (Table 1) [34, 35]. Traditionally, the urinary environment was considered sterile; however, recent research has demonstrated the presence of a diverse microbial population in both healthy and diseases [154, 155]. In 2011, Siddiqui et al. [156] reported that the urinary microbiota in healthy women was predominantly composed to *Lactobacillus* species, similar to the vaginal microbiota, while women with urinary incontinence had a more diverse and less stable microbial community. Furthermore, Lewis et al. [157] identified a distinct microbial signature in men with symptomatic urinary tract infection. Additionally, Thomas-White et al. [158] reported that women with recurrent urinary tract infections had a higher prevalence of certain bacteria, such as *Escherichia coli*, compared to healthy controls, and that the bladder microbiota of women with recurrent urinary tract infections was less diverse and less stable over time. A recent cross-sectional study demonstrated that alterations in the urinary microbiota are correlated with the severity of overactive bladder symptom in patients with overactive bladder, suggesting that urinary dysbiosis may play a role in the deteriorations of functional bladder diseases [159]. Collectively, it is worth noting that the urinary microbiota may play a role in various urinary tract conditions, including urinary tract infections, intestinal cystitis, urinary incontinence, and kidney stones (Fig. 2) [160, 161].

To the best of our knowledge, there are currently no articles reporting alterations in bladder microbiota in patients with neuropsychiatric disorders. However, a study by Wu et al. [162] demonstrated negative correlations between the severity of depression and both richness (Chao1) and diversity (Shannon index) of urinary microbiota in patients with overactive bladder. Furthermore, Ren et al. [163] found that compared with healthy group, patients with BD exhibited significantly higher levels of betaine, glycerol, hippuric acid, indole sulfate, trimethylamine oxide, and urea in their urine samples, while the level of inositol was significantly lower. Given the role of microbiota in the production of these compounds [164–166], it is possible that alternations in bladder microbiota may contribute to the observed changes in the urine sample composition [167–169]. It is important to note that further research is needed to investigate the potential link between the bladder microbiota, neuropsychiatric disorders, and urine sample composition. Understanding these relationships could provide valuable insights into the role of the bladder microbiota in the context of neuropsychiatric disorders and urinary metabolites.

Notably, there is evidence suggesting a link between overactive bladder and psychiatric disorders such as depression and anxiety [170, 171]. A national cohort study conducted in Taiwan demonstrated significantly higher risk of depression and anxiety in patients with overactive bladder compared to those without overactive bladder [172]. Additionally, a study involving older Korean women (*n* = 3000) revealed a higher prevalence of depression, stress, and low self-esteem in women with urinary incontinence [173]. Moreover, a recent prospective UK cohort study found associations between mental health problems, stressful life evens, and new-onset urinary incontinence in primary school-age children [174]. These findings collectively suggest that disturbances in bladder function may contribute to mental health problems across different age groups, from children to elderly individuals. Given the emerging understanding of the role of bladder microbiota in bladder function [154, 155, 175], it is of great interest to investigate whether the bladder microbiota plays a role in both bladder function and psychiatric symptoms in patients with neuropsychiatric disorders. Further research in this area may provide valuable insights into the complex relationship between bladder function, neuropsychiatric disorders, and bladder microbiota.

## Vagina microbiota

**Fig. 5:**
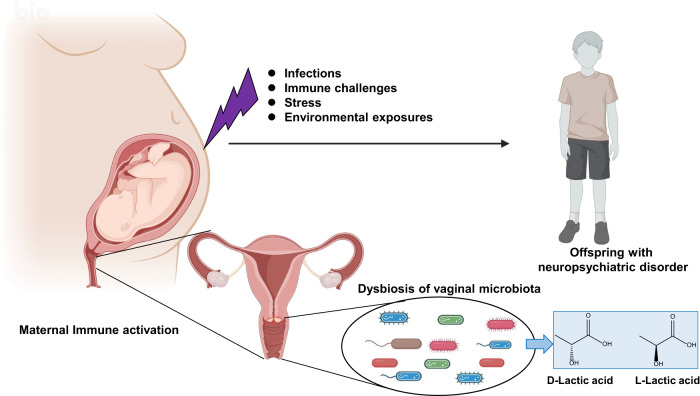
Potential role of vaginal microbiota in the development of neuropsychiatric disorders in offspring following maternal immune activation. The vaginal microbiota, particularly, the *Lactobacillus* species, plays several significant roles in maintaining vaginal health. There is an association between the composition of the vaginal microbiota and reproductive health, including the risk for spontaneous preterm birth. Maternal immune activation (MIA), which can result from a range of factors such as infections, immune challenges, stress, environmental exposures, has been linked with an increased risk of neuropsychiatric disorders in offspring. Dysbiosis of the vaginal microbiota due to MIA, along with subsequent disrupted immune responses, may foster the development of neuropsychiatric disorders in offspring. This imbalance in the vaginal microbiota might impact the synthesis of D- and L-lactic acid and other metabolites, leading to changes in pH and the immune system in the vagina. Furthermore, an imbalance in the vaginal microbiota may influence mental health issues in women throughout their lives. Part of the figure was designed using resources from Biorender.com.

The vaginal microbiota is a dynamic ecosystem that plays a role in women’s health [176–178]. The dominant bacteria in the vaginal microbiota are species of *Lactobacillus* (Table 1) [34, 35]. *Lactobacillus* species play several important roles in vaginal health, including the production of lactic acid, which helps maintain an acidic pH in the vagina (typically around 3.5–4.5), creating an inhospitable environment for pathogens [179, 180]. In women with a dominant microbiota *Lactobacillus spp*., the concentration of lactic acid is approximately 110 mM, acidifying the vagina to a pH of ~3.5 [178–180]. Two enantiomers of lactic acid exist. L-lactic acid is a common compound of human metabolism; however, both D- and L-lactic acid can be produced by certain strains of the microbiota or through unknown metabolic pathways (Fig. 5). D-lactate dehydrogenase is an enzyme that converts D-lactic acid to pyruvate [178, 181]. Currently, it remains unclear how *Lactobacillus* species in the vagina produce both enantiomers of lactic acid. Further studies on the quantification of two enantiomers of lactic acid in the vagina are needed.

The composition of the vaginal microbiota can be influenced by various factors, including different stages of a woman’s life (e.g., puberty, menstruation, pregnancy, menopause), hormonal status, sexual activity, hygiene practices, and underlying health conditions [176, 178]. There is a reported link between the profile of the vaginal microbiota and the incidence and prevalence of human papillomavirus [182]. It’s well established that women can encounter a variety of mental health issues, including depression, anxiety, postpartum depression, eating disorders, premenstrual dysphoric disorder, and perimenopausal depression. Although the direct relationship between the vaginal microbiota and neuropsychiatric disorders is not yet well understood, there are several potential ways in which the vaginal microbiota could influence these disorders. The vaginal microbiota plays a crucial role in maintaining a healthy immune system in the female reproductive tract. An imbalance in the vaginal microbiota can lead to inflammation and immune dysfunction, as well as changes in hormones and microbiota-derived metabolites, contributing to the development and progression of neuropsychiatric disorders (Fig. 5) [176, 178].

The successful application of fecal microbiota transplantation (FMT) has opened new avenues for the development of vaginal microbiota transplantation (VMT) [183–187]. VMT is an emerging clinical procedure designed to reestablish a balanced vaginal microbiota by transferring it from a healthy donor to a patient with vaginal microbiota dysbiosis [188, 189]. In 2019, Lev-Sagie et al. [190] reported the therapeutic effectiveness of VMT in women suffering from persistent and recurrent bacterial vaginosis, following pretreatment with antibiotics to eliminate pathogens. A recent case study demonstrated a successful VMT procedure, where donor strain engraftment was verified. This was followed by a successful pregnancy and childbirth after a series of previous late pregnancy losses or stillbirths [191]. If an imbalanced vaginal microbiota is found to be associated with mental health issues in women, VMT could emerge as a potential therapeutic option.

Emerging evidence suggests that maternal immune activation can increase the risk of neuropsychiatric disorders in offspring, including ASD, schizophrenia, and other neurodevelopmental and neuropsychiatric disorders [192, 193]. Various factors, such as infections, immune challenges, stress, and environmental exposures during pregnancy, can trigger an immune response in the mother (Fig. 5). There are significant concerns regarding the potential impact of maternal infection of COVID-19 on the development of neuropsychiatric disorders in offspring [194–197]. It remains unclear whether maternal immune activation can affect the vaginal microbiota in pregnant women. However, there is plausible that maternal immune activation could impact the vaginal microbiota, potentially leading to the development of neuropsychiatric disorders in offspring (Fig. 5). A recent meta-analysis using rodent studies showed that perinatal maternal microbiota disturbance is transmitted to the offspring, negatively impacting behavioral parameters related to neuropsychiatric disorders [198]. Understanding the relationship between maternal immune activation and the vaginal microbiota in the context of neuropsychiatric disorders in offspring is crucial for early detection, prevention, and intervention strategies. Further research is needed to uncover the underlying biological mechanisms, identify potential biomarkers (e.g., vaginal bacteria, and metabolites), and develop effective interventions (e.g., VMT) [189] to mitigate the impact of maternal immune activation on neurodevelopment and reduce the risk of neuropsychiatric disorders in the offspring of mothers who experience immune activation during pregnancy.

## Conclusion remarks and future directions

As outlined earlier, multiple lines of evidence indicate that dysbiosis in the gut microbiota could contribute to the onset and progression of various psychiatric and neurodegenerative disorders. However, research on the host microbiota in other organs, such as mouth, nose, lung, skin, bladder, and vagina, remains limited. Investigating the microbiota in these other organs is essential, as they can interact with the gut microbiota in the GI tract through inflammatory and immune system pathways (Fig. 2) [31, 34, 41, 42]. Future comprehensive studies aim to uncover the biological mechanisms by which alterations in host-microbiota contribute to the development and progression of neuropsychiatric disorders. Another key goal is to identify reliable biomarkers linked to host microbiota that could facilitate early detection, diagnosis, and monitoring of these conditions.

Moreover, there is significant potential for microbiota-targeted interventions (e.g., plant-based diet, probiotics, prebiotics, symbiotics, microbiome-derived metabolites, and microbiota transplantation) in the treatment and prevention of neuropsychiatric disorders. A recent double-blind, placebo-controlled study demonstrated that adjunctive treatment of multiple probiotics significantly reduced depression scores in MDD patients, without causing serious adverse effects [199]. While the use of FMT in treating neuropsychiatric disorders is on the rise [185–187], microbiota transplantation in other organs, such as VMT, could also offer therapeutic possibilities. Longitudinal studies that monitor changes in host microbiota and their correlation with neuropsychiatric disorders will be essential. Ultimately, translating microbiota-based interventions into clinical practice will be a critical advancement in the field [200].

The human microbiota, consisting of trillions of microorganisms residing both within and on our bodies, interacts intricately with approximately 20,000–25,000 genes in each individual. This complex interplay between the host microbiota and human genes plays a crucial role in our health and disease susceptibility. In conclusion, the future research focusing on the role of the host microbiota in neuropsychiatric disorders offers significant promise for elucidating the complex relationships between the microbiota in various tissues and the brain. This research has the potential to open new avenues for diagnostic methodologies and innovative therapeutic strategies for these disorders.
